# Dietary Habits and Musculoskeletal Pain in Statin and Red Yeast Rice Users: A Pilot Study

**DOI:** 10.3390/ejihpe11040085

**Published:** 2021-09-29

**Authors:** Anna Raguzzini, Elisabetta Toti, Maura Palmery, Mohamed M. Abdel-Daim, Ilaria Peluso

**Affiliations:** 1 Research Centre for Food and Nutrition, Council for Agricultural Research and Economics (CREA-AN), 00142 Rome, Italy; anna.raguzzini@crea.gov.it (A.R.); elisabetta.toti@crea.gov.it (E.T.); 2 Department of Physiology and Pharmacology “V. Erspamer”, Sapienza University of Rome, 00185 Rome, Italy; maura.palmery@uniroma1.it; 3 Pharmacy Program, Department of Pharmaceutical Sciences, Batterjee Medical College, Jeddah 21442, Saudi Arabia; abdeldaim.m@vet.suez.edu.eg; 4 Pharmacology Department, Faculty of Veterinary Medicine, Suez Canal University, Ismailia 41522, Egypt

**Keywords:** food-drug interaction, lifestyle, nutraceutical-drug interaction, physical activity

## Abstract

(1) Background: Diet and statins are commonly used to treat high cholesterol (CHOL) levels. (2) Aim: To compare adherence to Mediterranean diet (Med-D), orthorexia nervosa (ON), and musculoskeletal pain in individuals in treatment with statins metabolized by CYP3A4, not metabolized by CYP3A4 or red yeast rice (RYR, containing monacolin K: MON-K). (3) Methods: starting from 80 individuals, after the exclusion of those with other causes of possible pain, 56 individuals were selected and divided into three groups according to the type of statin (CYP3A4, NO-CYP3A4 and MON-K). Adherence to the Med-D was evaluated with the MEDScore and a sub-score was calculated for fruit and vegetables consumption (MEDScore-FV). ON and musculoskeletal pain were assessed with the ORTO-15 and with the Nordic Musculoskeletal questionnaires, respectively. A retrospective analysis of CHOL decrease after treatment was conducted. (4) Results: CHOL levels were lower in CYP3A4 and NO-CYP3A4 after treatment (182.4 ± 6.3 and 177.0 ± 7.8 mg/dL, respectively), compared with MON-K (204.2 ± 7.1 mg/dL, *p* < 0.05). MON-K and CYP3A4 groups had a high prevalence of reported knee pain (33.3% and 18.8%, respectively) than NO-CYP3A4 group (0%, *p* < 0.05). A high percentage of individuals in MON-K take supplements and nutraceuticals (87.5%), whereas MEDScore-FV was higher in CYP3A4 (9.4 ± 0.2) compared to NO-CYP3A4 (7.6 ± 0.5, *p* < 0.05). (5) Conclusions: This study suggests that individuals receiving treatment with statins and RYR should be monitored from the perspective of plant foods’ consumption and nutraceutical use, to prevent musculoskeletal pain.

## 1. Introduction

The additive cholesterol (CHOL)-lowering effects of statins’ treatment and Mediterranean diet (Med-D), rich in fruits, vegetables, monounsaturated and omega 3 fatty acids, are known since 2002 [1]. Although in the ATTICA study [2] a high adherence to Med-D reduced cardiovascular disease (CVD) risk independently of statin use, in the Moli-sani study [3] the association of Med-D and statin decreased the mortality more than Med-D or statin use alone in patients at risk of CVD. Besides, Med-D in combination with red yeast rice (RYR) reduced low-density lipoproteins (LDL) in individuals with statin intolerance [4]. RYR, obtained by the traditional fermentation of cooked rice kernels with a Monascaceae mold, *Monascus purpureus* [5], by solid-state culture method [6] or by liquid-state culture method [6], contains monacolin K, a weak reversible inhibitor of 3-hydroxy-3-methyl-glutaryl-coenzyme A reductase, the rate-limiting enzyme of the mevalonate pathway of cholesterol synthesis [7]. Although citrinin (CIT), a nephrotoxic and hepatotoxic mycotoxin, is produced by *Monascus* spp., it has been reported that dietary supplements contained CIT below the established limit of detection [8]. On the other hand, monacolin K is often associated with berberine in commercial nutraceutical, but scarce standardization and discrepancy between the doses of bioactive molecules reported by the manufacturers and the amounts resulting from analysis of the same products were reported [9]. The European Food Safety Authority (EFSA) Panel on Food Additives and Nutrient Sources added to Food (ANS) in 2018 concluded that exposure to monacolin K from RYR could lead to severe adverse effects on musculoskeletal system [10]. Authorities stated that food supplements containing RYR were of significant safety concern at the dosage of 10 mg/day and that individual cases of severe adverse reactions have been reported for monacolins from RYR at intake levels as low as 3 mg/day [10]. However, Cicero et al. recently concluded that the risk related to 3 to 10 mg/day monacolin K is minimal (mild myalgia in previously severely statin-intolerant subjects) [7]. Despite a meta-analysis suggested an improvement of moderate hypercholesterolemia by RYR without increased incidence of muscular adverse effects [11], in clinical practice experience, 1/18 patients with statin-associated muscle symptoms had side effects after nutraceutical treatment [12]. Recently, most reported adverse drug reactions for RYR were labelled musculoskeletal and connective tissue disorders (*n* = 64 among 94 case reports) [13]. Monacolin K is structurally identical to lovastatin [11], therefore it is metabolized by cytochrome P450 (CYP)3A4 isoenzyme, such as lovastatin, atorvastatin and simvastatin, whereas rosuvastatin and pravastatin (not susceptible to CYP inhibition) could be less prone to food-drug interactions [14,15]. However, organic anion transporters (OAT)-mediated interactions on rosuvastatin pharmacokinetic have been reported for honey flavonoids [16] and epigallocatechin gallate [17]. Vaquero et al. [15] pointed out that studies on the effect of statins in patients consuming a Med-D are necessary to assure the correct prescription. Relationships between adherence to Med-D and orthorexic behavior [18] and between orthorexia nervosa (ON) and a high level of physical activity [19] have been reported. Despite statins having a safety profile, many patients reported muscle symptoms which contribute to drug discontinuation and reducing physical activity [20]. In this context, a recent study found that sitting time was associated with musculoskeletal symptoms [21]. From that, our hypothesis was that patients in treatment with statins metabolized by CYP3A4 or mon-K could report more pain when consuming plant food potential interfering with phase I metabolism and can be more aware of their food consumption, more adherent to Med-D, and sedentary. Therefore, in this study we aimed to compare adherence to Med-D, ON [22] and self-reported musculoskeletal pain in statin and RYR users. Moreover, the reduction in CHOL levels (retrospectively) and potential confounders, including use of other drugs, physical activity and sitting time, were evaluated.

## 2. Materials and Methods

This retrospective observational study was approved by the Ethics Committee for Human Non-Clinical Research of the Department of Physiology and Pharmacology “V. Erspamer”, “Sapienza” University of Rome (Approval Date: 18 April 2018) and all procedures involving human subjects, complied with the Declaration of Helsinki as revised in 2000. Adult individuals were recruited (April 2019–February 2020) by pharmacies and verbal disclosures.

During the selection of volunteers, the following inclusion criteria were applied:

- statins or RYR use;
- consent to furnish data from questionnaire;
- consent to furnish analysis report.

A total of 80 volunteers were enrolled and all participants provided a signed informed consent. Information submitted through the questionnaires were used to characterize the subjects and included body mass (BM), height, medical history, postmenopausal state and previous pregnancy and/or spontaneous abortion (for women), use of drugs and supplements, smoking habits and consumption of alcoholic beverages, cocoa, coffee and tea. The body mass index (BMI) was calculated dividing BM (in kg) by squared height (in meter). Primary outcomes were the degree of adherence to the Med-D, ON and the musculoskeletal symptoms. Adherence to the Med-D was calculated through the MEDScore proposed by Panagiotakos [23]. Besides a sub-score for fruit and vegetables was calculated (MEDScore-FV: range 0–10). The ORTO-15 score was assessed according to Donini et al. [22] and also ORTO 12, ORTO-11, ORTO-9 and ORTO-7 were calculated [24].

The musculoskeletal pain was assessed through the Nordic Musculoskeletal Questionnaire (NMQ) [25], previously used to evaluate the effects of statins [26] and musculoskeletal symptoms in workers [21].

Daily sitting time (minutes/day), as well as weekly time spent in walking and in moderate and high intensity activities, were self-reported by the respondents when answering specific items in the International Physical Activity Questionnaire (IPAQ) as previously described [21].

The individuals enrolled in the study were asked to provide diagnostic reports (pre- and post-treatment) and the reduction in CHOL levels was calculated based on the blood test results provided by the volunteers.

**Figure 1:**
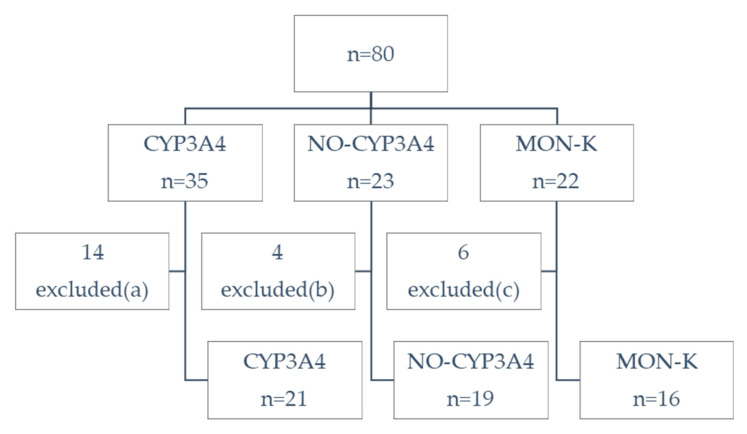
Flow diagram. CYP3A4: volunteers in treatment with lovastatin, atorvastatin or simvastatin; NO-CYP3A4: volunteers in treatment with rosuvastatin or pravastatin; MON-K: volunteers in treatment with monacolin K (red yeast rice, RYR). Excluded for potential confounders (other causes of pain): (a) 9 injury in the last 12 months, 4 arthritis, 1 in treatment with both atorvastatin and rosuvastatin (b) 4 injury, (c) 5 injury, 1 arthritis.

Data of the 80 enrolled individuals allowed us to divide the sample into three groups: CYP3A4 (in treatment with lovastatin, atorvastatin or simvastatin), NO-CYP3A4 (in treatment with rosuvastatin or pravastatin) and MON-K (in treatment with RYR). After the exclusion of individuals with other causes of possible pain (Figure 1), 56 individuals were analyzed.

### Statistical Methods

Categorical variables were expressed as percentages and significance assessed by the χ^2^ test. Continuous variables were expressed as means and standard error of the mean (SEM). Results passing Equal Variance or Normality test (Shapiro–Wilk) were analyzed by analysis of variance (ANOVA) and Student Newman–Keuls method, others by Kruskal–Wallis one-way analysis of variance on ranks and Dunn’s method.

## 3. Results

Table 1 shows the characteristics of the study sample according to statins or RYR use. Compared to RYR (MON-K) users, statins (CYP3A4 and NO-CYP3A4) users were older (*p* < 0.001). There were no statistically significant differences by gender and approximately two-thirds of the subjects were women in the group CYP3A4 and MON-K, while in the NO-CYP3A4 users were close to one-third. Menopausal state was present in 71.4% of CYP3A4 women, 85.7% of the NO-CYP3A4 and 45.4% of MON-K.

No statistically significant differences were found between the three groups in terms of smoking habits, BMI, blood pressure and heart rate (Table 1), in any case a high prevalence of the subjects declared to take antihypertensive (Table 1).

A higher dose and a longer duration of treatment has been observed in CYP3A4 and NO-CYP3A4 users compared with MON-K (Table 1).

Accordingly, the retrospective analysis of CHOL levels revealed that MON-K was less efficacious than statins (Table 1).

The results related to the use of other drugs show that MON-K users take fewer drugs (as they are younger than CYP3A4 and NO-CYP3A4), but more nutraceuticals (Table 1). In particular, 81% of the products with RYR also contain other lipid-lowering nutraceuticals like policosanols, berberine and polyunsaturated ω-3 fatty acids, at the same time containing vitamins and minerals (56% and 38% of the products, respectively). On the other hand, non-significant difference was found in the prevalence of the use of anti-inflammatory/analgesic drugs (Table 1).

Table 2 shows the self-reported musculoskeletal pain in different anatomical regions for statin users compared to RYR users during the last 7 days. The prevalence of pain was higher among patients in treatment with statins metabolized by CYP3A4 compared with the other groups (NO-CYP3A4 and MON-K), but also individuals in the MON-K group reported lower extremity pain (Table 2).

Few subjects practiced a sport especially in group NO-CYP3A4, while 31% of the subjects in group MON-K practiced at least one sport (Table 3). However, differences in sport practice, physical activity, walking and sitting time did not reach significance (Table 3).

Table 3 presents the results relating to the dietary habits of the sample. There were no statistically significant differences for adherence to Med-D (MEDScore), whereas individuals in the CYP3A4 group had higher MEDScore-FV compared to NO-CYP3A4.

We observed a non-significant higher prevalence of consumers of fruit juice and tea in MON-K group (Table 3). On the other hand, there were no statistically significant differences for tea, chocolate, coffee, and alcoholic beverages consumption (Table 3). Furthermore, no significant differences in ON were observed among groups (Table 3).

## 4. Discussion

The prevalence of pain in any region was 66.7%, 42.1% and 68.8% for CYP3A4, NO-CYP3A4 and MON-K, respectively. This is in agreement with our hypothesis and with the conclusion of EFSA Panel [10] and with previous case reports [12,13]. The body regions with the higher prevalence of symptoms in the CYP3A4 group were wrist/hand, knees and ankle/feet. Although these body parts are not the mainly affected muscles from statin-induced myopathy, a case of statin-induced bilateral foot myopathy has been reported [28] and it has been proposed that the decrease in lower extremity muscle-strengthening activities could be related to the increase in muscle pain associated with statin therapy [29]. Knee pains were also reported by MON-K users with a high prevalence compared to individuals in the NO-CYP3A4 group. However, low extremity pain can be due to other causes. A higher percentage of construction workers who practice moderate or vigorous leisure-time physical activity (LTPA), including walking, bicycling, hockey, weight lifting and gardening, reported lower extremity pain (i.e., ankle, knee) compared with those who did not engage in either LTPA (57% and 21%, respectively) [30]. Whereas increasing steps per day reduced pain in adults with fibromyalgia [31] and with musculoskeletal diseases [32], the association of statins and exercise is controversial [33,34,35,36,37] and a case of rhabdomyolysis-induced by atorvastatin and strenuous exercise has been reported [38]. The development of statin-related myopathy may be enhanced by acute and chronic physical exercises. According to the European Society of Cardiology (ESC) and the European Atherosclerosis Society (EAS), individuals affected by dyslipidemias should be encouraged to practice regular (30 min/day) moderate physical activity, but they have to must to pay attention to myopathy and creatine kinase (CK) elevation related to statin treatment [39]. It has been suggested that obsessive features of sport activities (guilt over skipping training, counting calories during training) play an important role in ON [40]. ON was often seen in men in exercise science studies in combination with a high level of physical activity and a high degree of self-reported pain [19]. Others suggested that obsessive features of sport activities (guilt over skipping training, counting calories during training) play an important role in ON [40]. On the other hand, ON has a bidimensional nature, including the pathological preoccupation for food and the healthy orthorexia, which could possibly be seen as a protective behaviour [41,42,43]. For example, ON symptoms were positively correlated to physical activity, fruit and vegetable consumption, body appreciation, and life satisfaction, in Chinese elderly [44]. In the present study prevalence of ON was about double when cut-off of 40 was applied, compared to cut-off of 35, as assessed with ORTO-15, with no differences among groups and there were no significant differences also for sub-scores ORTO-12, ORTO-11, ORTO-9 and ORTO-7. In other studies ON was associated with the use of dietary supplements [45,46]. Although no significant differences in ON were observed, individuals in the MON-K group used more nutraceuticals than other groups. In this context, omega-3 polyunsaturated fatty acids (omega-3 PUFA) supplementation has been suggested for preventing or treating statin myopathy [47] and for inflammation-mediated pain in sarcopenic elderlies [48]. Although a meta-analysis found that supplementation with omega-3 PUFA did not result in a clinically relevant reduction of muscle soreness after eccentric exercise [49], omega-3 PUFA are among the antinociceptive and analgesic natural compounds [50]. The analgesic effect can mask the reported pain due to the statin-induced myopathy. In the present study, the use of nutraceuticals is statistically significant, with a prevalence of 81.3% in MON-K users. MON-K group includes more individuals who take omega 3 fatty acids, and this could be a confounding factor for self-reported pain. On the other hand, the prevalence of oral analgesic use, in CYP3A4 (42.8%) and NO-CYP3A4 (42.1%), was similar to that previously reported in individuals with musculoskeletal disorders with lipid-lowering drug use (41.0%) [26]. On the contrary, in the MON-K group the prevalence of analgesic use (18.8%) was comparable to that found in the subjects with lipid-lowering drug use without musculoskeletal disorders (19.0%) [26].

However, a limitation of this study is that volunteers in the MON-K group were younger compared to those in the other groups. On the other hand, MEDScore-FV was higher in the CYP3A4 group, compared to the NO-CYP3A4. This result prevented us from investigating the potential interactions between flavonoid-rich foods and statins [7].

In addition to fruit juices, particularly grapefruit juice known to inhibit CYP3A4 by reducing the metabolism of simvastatin, lovastatin and atorvastatin [14,15], tea flavanols can interfere with the pharmacokinetics of drugs in humans, by interaction with CYP3A4 and with phase III transporters of the drug detoxifying system, mainly the multidrug resistance 1 (MDR1) [51], OAT [17,51], and organic cation transporters (OCT) [51]. However, the consumption of fruits and vegetables [52,53], rich in antioxidant flavonoids, as well as Med-D and physical activity [53], can reduce musculoskeletal pain. Med-D can reduce chronic pain [54], and a high adherence to the Med-D was associated with pain improvement in older adults recruited in the Seniors-ENRICA-1 and Seniors-ENRICA-2 cohorts [55]. Furthermore, the health effects of Med-D are well documented [1,2,3,4] and relationships between adherence to Med-D and ON [18] have been reported. However, in this pilot study we did not observe the hypothesized differences among groups in the adherence to Med-D and in ON. Besides, the hypothesized differences among groups in sport practice, physical activity, walking and sitting time did not reach significance. The major limitation of our study is the relatively limited sample size and further investigation would be desirable. Moreover, non-probabilistic sampling makes our preliminary results not applicable to general population.

## 5. Conclusions

In line with the recommendation of other authors [15], this pilot study suggested that individuals receiving treatment with statins and RYR should be monitored from the perspective of plant foods’ consumption and the use of nutraceuticals that can reduce pain or interfere with statins’ pharmacokinetic, to prevent musculoskeletal pain. Moreover, self-reported pain should be evaluated in conjunction with physical activity level [20,21]. Despite statins and RYR having a safety profile [11], our results confirm the needs of further investigation to finally describe the safety profile of these drugs metabolized by CYP3A4. Appropriate information about the potential adverse effects of RYR on musculoskeletal system should be provided to clinicians and patients also in order to submit suspected adverse effects to agencies and continuous monitoring the safety of “natural” dietary supplements.

## Figures and Tables

**Table 1:** Characteristics of individuals.

	CYP3A4	NO-CYP3A4	MON-K	*p*
Women%	57.1	31.6	68.8	0.075
Menopausal state % (within women)	71.4	85.7	45.4	0.179
Age years	66.0 ± 1.9	65.5 ± 2.3	51.5 ± 2.6	<0.001
BMI kg/m^2^	25.5 ± 0.7	24.9 ± 0.6	24.4 ± 1.1	0.606
SBP mmHg	126.3 ± 2.6	129.6 ± 2.7	123.9 ± 2.1	0.262
DBP mmHg	75.1 ± 1.9	80.0 ± 1.8	77.1 ± 1.3	0.129
HR beats/m	68.4 ± 2.4	71.6 ± 1.2	76.5 ± 3.1	0.075
Smokers %				0.653
Habitually	9.5	10.5	6.3	
Occasionally	0.0	10.5	6.3	
CHOL mg/dl				
Pre-treatment	252.8 ± 8.8	252.8 ± 9.7	242.9 ± 6.3	0.664
Post-treatment	182.4 ± 6.3	177.0 ± 7.8	204.2 ± 7.1	0.028
Delta-CHOL	−70.4 ± 8.1	−75.7 ± 14.8	−38.6 ± 9.5	0.057
Statin treatment				
Dose mg/d	19.8 ± 2.1	19.2 ± 2.8	8.5 ± 1.1	0.001
Duration years	7.0 ± 1.4	4.7 ± 0.8	1.4 ± 0.3	0.002
Other drugs %				
Other lipid lowering	9.5 ^(1)^	21.0 ^(1)^	0	0.130
Hormones	4.8 ^(2)^	10.5 ^(2)^	18.7 ^(3)^	0.351
BP lowering	57.1 ^(4)^	36.8 ^(5)^	18.7 ^(6)^	0.076
Anti-platelets/anti-coagulant	23.8 ^(7)^	26.3 ^(8)^	0	0.099
Glucose lowering	23.8 ^(9)^	15.9 ^(10)^	0	0.134
Anti-inflammatory/analgesic %				
Oral drugs	42.8 ^(11)^	42.1 ^(11)^	18.8 ^(11)^	0.245
Topical	33.3 ^(12)^	36.8 ^(12)^	31.2 ^(12)^	0.939
Nutraceuticals %	0	5.2 ^(13)^	87.5 ^(14)^	<0.001

CYP3A4: volunteers in treatment with lovastatin, atorvastatin or simvastatin; NO-CYP3A4: volunteers in treatment with rosuvastatin or pravastatin; MON-K: volunteers in treatment with monacolin K (red yeast rice, RYR); SBP: systolic blood pressure; DBP: diastolic blood pressure; HR: heart rate; Delta-CHOL: Cholesterol variation after treatment. ^(1)^ ezetemibe; ^(2)^ levothyroxine; ^(3)^ levothyroxine, norethisterone acetate; ^(4)^ amlodipine, atenolol, bisoprolol, candesartan, irbesartan, mesartan, metoprolol tartrate, nebivolol, rampiril, perindopril, valsartan, zofenopril calcium salt; ^(5)^ atenolol, bisoprolol, doxazosin, enalapril, rampiril, valsartan; ^(6)^ rampiril, olmesartan; ^(7)^ apixaban, clopidogrel, digitalis glycoside; ^(8)^ clopidogrel, acetylsalicylic acid; ^(9)^ dulaglutide, insulin, metformin; ^(10)^ metformin; ^(11)^ tapentadol, cortisone; ^(12)^ cortisone; ^(13)^ berberine, polyunsaturated ω-3 fatty acids; ^(14)^ policosanols, berberine, β-Sitosterol, octosanol, polyunsaturated ω-3 fatty acids.

**Table 2:** Musculoskeletal pain.

	CYP3A4	NO-CYP3A4	MON-K	*p*
Number of painful—last 7 days	2.1 ± 0.5	1.0 ± 0.4	1.2 ± 0.3	0.105
Pain last 7 days %	66.7	42.1	68.8	0.085
Neck	28.6	15.8	12.5	0.416
Shoulders	19.0	15.8	18.8	0.959
Elbows	9.5	5.3	0	0.444
Wrists/hands	23.8	5.3	0	0.043
Dorsal	23.8	21.1	12.5	0.680
Lumbar	28.6	31.6	43.8	0.605
Hip/thighs	14.3	10.5	0	0.306
Knees	33.3	0	18.8	0.023
Ankles/feet	33.3	0	6.3	0.006

CYP3A4: volunteers in treatment with lovastatin, atorvastatin or simvastatin; NO-CYP3A4: volunteers in treatment with rosuvastatin or pravastatin; MON-K: volunteers in treatment with monacolin K (red yeast rice, RYR). Categorial variables were expressed as percentages (χ^2^ test). Continuous variables were expressed as means with standard error mean (SEM).

**Table 3:** Physical activity and dietary habits.

	CYP3A4	NO-CYP3A4	MON-K	*p*
Sports practice %	23.8 ^(1)^	15.8 ^(2)^	31.3 ^(3)^	0.557
Physical activity min/week				
Moderate intensity	216.7 ± 59.1	165.0 ± 64.1	162.3 ± 67.7	0.781
High intensity	96.5 ± 42.8	66.1 ± 46.0	131.1 ± 67.1	0.690
Walking min./week	288.3 ± 58.4	180.8 ± 44.4	281.6 ± 54.9	0.295
Sitting min./day	197.1 ± 31.0	182.8 ± 25.7	226.9 ± 37.2	0.717
MEDScore	33.7 ± 1.1	33.4 ± 1.1	36.4 ± 1.3	0.190
MEDScore-FV	9.4 ± 0.2 *	7.6 ± 0.5 *	8.7 ± 0.5	0.009 *
Fruit juices ml/day	17.8 ± 9.8	19.7 ± 10.7	39.1 ± 15.0	0.396
Tea ml/day	77.4 ± 26.6	65.8 ± 20.0	117.2 ± 21.3	0.335
Dark chocolate/week	2.3 ± 0.6	1.0 ± 0.3	2.1 ± 0.7	0.145
Wine/week	2.7 ± 0.7	2.3 ± 0.7	1.1 ± 0.5	0.235
Beer/week	0.4 ± 0.1	0.3 ± 0.1	0.5 ± 0.3	0.618
Coffee/day	1.9 ± 0.3	1.7 ± 0.2	1.7 ± 0.3	0.666
ORTO-15	36.2 ± 0.9	38.2 ± 0.8	36.9 ± 1.3	0.350
<40	71.4%	52.6%	68.7%	0.421
<35	38.1%	10.5%	25%	0.132
ORTO-12 ^(4)^	29.6 ± 0.7	30.7 ± 0.7	30.9 ± 1.2	0.499
ORTO-11	26.3 ± 0.7	27.3 ± 0.7	27.4 ± 1.0	0.541
<25	38.1%	15.8%	25.0%	0.277
ORTO-9	24.7 ± 0.8	26.1 ± 0.8	23.9 ± 0.9	0.191
<26.7	66.7%	47.4%	68.7%	0.339
ORTO-7	17.9 ± 0.4	18.8 ± 0.5	19.4 ± 0.8	0.195
<20	61.9%	68.4%	50.0%	0.534

CYP3A4: volunteers in treatment with lovastatin, atorvastatin or simvastatin; NO-CYP3A4: volunteers in treatment with rosuvastatin or pravastatin; MON-K: volunteers in treatment with monacolin K (red yeast rice, RYR). ^(1)^ gymnastics, swimming, skating; ^(2)^ jogging, trekking, dancing, cycling; ^(3)^ gymnastic, swimming, dancing. MEDScore: adherence to the Mediterranean diet. ORTO: scores of orthorexia nervosa. ^(4)^ No cut-off values [27]. Categorial variables were expressed as percentages (χ^2^ test). Continuous variables were expressed as means with standard error mean (SEM). Student-Newman-Keuls: multiple comparison * = *p* < 0.05

## Data Availability

Individuals’ data availability is restricted by the Ethics Committee to protect privacy.
